# An Unusual Diagnosis for an Infant Presenting with Olive-Shaped Abdominal Mass

**DOI:** 10.1155/2023/6924037

**Published:** 2023-09-25

**Authors:** Ghada Maher Abdulaziz, Khalid Maher Mohamed, Ayat Hassan Zilai, Asmaa Ibrahim Attieh, Eman Abdulmohsen Alhammad, Abdullah Yahya Akkam

**Affiliations:** ^1^Pediatric Emergency Department, King Saud Medical City, Riyadh, Saudi Arabia; ^2^King Saud Medical City, Riyadh, Saudi Arabia

## Abstract

A 3-month-old male infant was bought to the emergency department with almost 3 weeks of projectile, bilious emesis after each feed. On presentation, he was cachectic and severely dehydrated and had a palpable olive in the epigastric region. Hypertrophic pyloric stenosis was excluded by abdominal ultrasound. The barium meal demonstrated a massively distended stomach, absence of distal gas, and triple bubble sign. Given the radiological findings, the patient underwent an emergency exploratory laparotomy and a congenital duodenal web (CDW) was discovered. While the clinical picture suggested the diagnosis of HPS, barium meal eventually revealed congenital duodenal web. To our knowledge, no other cases of palpable olive mass as the presenting sign of proximal bowel obstruction have been published.

## 1. Introduction

Congenital duodenal web is a reported cause of intestinal obstruction in neonates, and its symptomatic incidence is 1 : 10,000 to 1 : 40,000 [1]. Presentation beyond the neonatal period poses a diagnostic challenge and necessitates a high index of suspicion to diagnose such anomalies. We present an unusual presentation of the duodenal web in a 3-month-old boy who presented with chronic vomiting and visible peristaltic waves with an olive-shaped abdominal mass, mimicking hypertrophic pyloric stenosis.

## 2. Case Presentation

A 3-month-old male infant presented with 20-day history of bilious and projectile vomiting after each feed with substantial weight loss and lethargy. He is full-term with a birth weight of 3 kg (50^th^ percentile weight for age), a product of consanguineous healthy parents, had an uneventful prenatal history with a normal antenatal ultrasound, and is developmentally age appropriate.

The patient was admitted twice in different hospitals for fluid therapy. In his last admission almost 5 days prior to his current presentation, he had an abdominal ultrasound and hypertrophic pyloric stenosis was suspected. The parents decided to defer the pyloromyotomy to get a second medical opinion.

On examination, the infant was cachectic, lethargic, and severely dehydrated. He had multiple cautery marks on his abdomen performed by a traditional healer (Video 1). His weight upon admission was 2.9 kg (below the 3^rd^ percentile weight for age). He had a scaphoid abdomen and visible peristalsis transversely across the epigastrium from the infant's left to right (“golf ball sign”) (Videos 2 and 3). Test feeding demonstrated the classic olive sign upon palpation (Videos 2 and 3).

**Figure 1:**
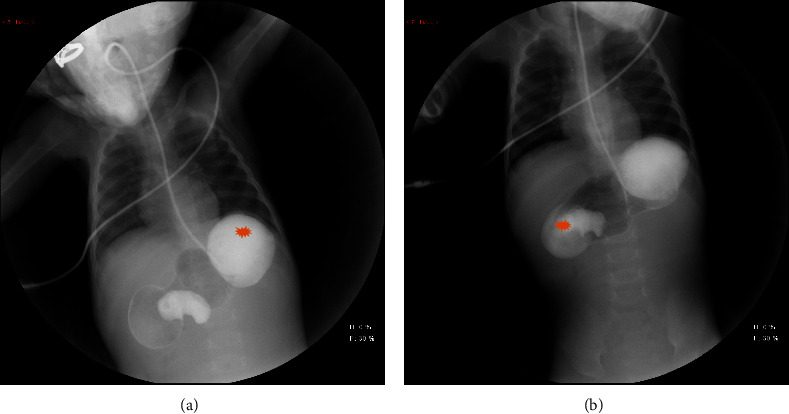
(a) Fundus of the stomach. (b) Windsock sign, typical for duodenal web.

The hard pylorus mass was firm and nontender, measuring 1 to 2 cm in diameter in the right upper quadrant. There were bilateral reducible inguinal hernias. The rest of the examination was normal. Investigations carried out in our patient showed normal complete blood count (CBC) and serum chemistry values including potassium (K^+^ 4.3 mmol) and chloride (Cl^−^ 96 mmol/L) VBG were remarkable for uncompensated metabolic alkalosis ph: 7.54 pCO_2_: 35.7 mmHg cHCO_3_: 31.1 mmol/L cBase 7.4 mmol/L. Ultrasound of the abdomen showed evidence of a markedly distended stomach and the first part of the duodenum with no signs of pyloric stenosis. Barium meal revealed an abnormally distended stomach, paucity of distal gas, and triple bubble sign consistent with a diagnosis of congenital duodenal obstruction (Figures 1(a) and 1(b)). The patient was taken immediately to the operating room for exploratory laparotomy. The web was incised, and bilateral inguinal hernia repair was performed. Postoperative care included monitoring in the PICU. Upper GI series demonstrated no leakage. Total parenteral nutrition (TPN) was provided until full recovery was achieved. The patient was discharged in a good clinical condition with follow-up appointments with paediatric surgery, general pediatrics, and dietician; however, he did not show up.

## 3. Discussion

Congenital duodenal anomalies are rare obstructive lesions [2] that originate from fetal defects in the early embryologic development of the foregut, with an incidence rate of approximately in 2,500 to 10,000 births [3, 4].

Clinical features of congenital duodenal obstruction include early vomiting that is often postprandial and bilious [3]. Differential diagnostic considerations vary given the site of anatomic defects that may be complete or partial, including annular pancreas, duodenal web, duodenal atresia, or duodenal stenosis. Congenital duodenal web (CDW) is a rare cause of intestinal obstruction with an incidence of 1 in 10,000 to 1 in 40,000 live births. Duodenal web (type I duodenal atresia) occurs due to failure of recanalization of the duodenal lumen during the 8^th^ to 10th week of gestation [5] The web comprised a thin layer of mucosa and submucosa only with absent muscularis, and it tends to cause the classical windsock deformity of the duodenal lumen on upper gastrointestinal contrast study [6].

Very few case reports of CDW have ever been described in the literature, particularly when the presenting infant has evidence of transverse peristaltic movement and a palpable olive. To the best of our knowledge, this is the first study to report such a presentation of CDW. Gilet et al. reported that several diseases presenting in the first 3 months of life with projectile vomiting may simulate hypertrophic pyloric stenosis (HPS), including pylorospasm and gastroesophageal reflux [7]. As in our case, an olive-shaped mass and transverse peristaltic movement in the epigastrium was elicited, despite normal serum electrolytes and he was later diagnosed with CDW. It is worth mentioning that vomiting in HPS should not be bilious, as bilious emesis is synonymous with intestinal obstruction. The vomiting in CDW may be bilious or nonbilious, depending on the level of obstruction. Bile-stained vomitus occurs in two-thirds of the cases of CDW that are distal to the ampulla of Vater[6, 8].

With the current advances in medicine, in utero detection of congenital duodenal obstruction through prenatal ultrasound is possible. Some prenatal markers include polyhydramnios, double bubble sign, and associated congenital anomalies. However, nearly half of the cases are not diagnosed until after birth and a third of them are identified later on in infancy. Therefore, clinicians must have a high index of suspicion when regurgitations persist, even low volume, in nonseptic neonates to avoid missing the diagnosis. We thereby stress the importance of taking a careful and complete history and performing a thorough physical examination as the foundation of diagnosis.

Delayed diagnosis of proximal bowel obstruction may lead to dehydration and clinically significant metabolic disturbances due to the ongoing loss of gastric secretion with persistent emesis. Thus, in view of the history, clinical examination, and venous blood gas findings, it is highly likely that this patient had been suffering from the consequences of the delayed diagnosis of congenital duodenal obstruction. Kilbride et al. reported that infants with delayed diagnosis of congenital duodenal obstruction have had poorer outcomes and that 12.5% of them died as a consequence of metabolic and pulmonary disturbances related to proximal bowel obstruction [9].

Finally, although proper assessment of vomiting is challenging to paediatricians, particularly in a nonseptic newborn, this report aims at reminding clinicians to be vigilant when emesis persists in the newborn, regardless of the volume, and that it is particularly vital to further evaluate those before discharge to avoid delays in operative intervention.

## 4. Conclusion

This case suggests that a palpable olive mass in the epigastric region may not be a distinct disease characteristic of hypertrophic pyloric stenosis but may instead be a presenting sign of proximal bowel obstruction. We also emphasize the importance of a thorough history taking and physical examination, followed by rapid and effective diagnostic techniques, such as point-of-care ultrasound. Moreover, it is vital that clinicians maintain an open diagnostic mind when approaching newborns with persistent vomiting as it could mimic less serious diseases.

## Data Availability

The data used to support the findings of this study are available from the corresponding author upon request.

## Supplementary Materials

Supplementary Materials Video 1: Multiple cautery marks on the abdomen, a traditional Arab healing practice that aims to relief symptoms. In skin cautery, a hot iron or coal is applied to the skin of the diseased body part for a few seconds in a symmetrical configuration, yielding permanent oval scars. Video 2 and 3: A firm olive-shaped mass resembling Gastric peristaltic wave is seen progressing across the abdomen.
